# Supplementary material for: Porous organic cages as synthetic water channels
Source: Nat Commun. 2020 Oct 1;11:4927. doi: 10.1038/s41467-020-18639-7 (PMC7530991; doi:10.1038/s41467-020-18639-7)
Supplement: Supplementary file 1 — Supplementary Information [file 41467_2020_18639_MOESM1_ESM.pdf]

Supplementary Information for

Porous organic cages as synthetic water channels

Yi Di Yuan[1], Jinqiao Dong[1], Jie Liu[1], Daohui Zhao[1], Hui Wu[2], Wei Zhou[2], Hui Xian Gan[1,3], Yen Wah Tong[1,3], Jianwen Jiang[1]*, Dan Zhao[1]*

Correspondence to: D.Z. (Dan Zhao, chezhao@nus.edu.sg, experiment) or J.J. (chejj@nus.edu.sg, simulation)

This supplementary file includes:

Supplementary Methods
Supplementary Figures 1 to 13
Supplementary Tables 1 to 5
Supplementary References

Other Supplementary Materials for this manuscript include the following:

Supplementary Movies 1 to 5
Supplementary Data 1

Supplementary Methods

Chemicals and equipment. All the chemicals were obtained from commercial suppliers and used without further purification. Lipids were purchased from Avanti Polar Lipids (Alabaster, AL). Gramicidin A, lucigenin dye, inorganic salts, Triton X–100, pyranine dye, and (4–(2–hydroxyethyl)–1–piperazineethanesulfonic acid) (HEPES) buffer were purchased from Sigma–Aldrich. Track-etched polycarbonate membranes and hand-held extrusion systems were purchased from Avanti Polar Lipids (Alabaster, AL). Fourier transform infrared spectroscopy (FTIR) data were obtained with a Bio–rad FTS–3500 ARX FTIR spectrometer. Ultraviolet–visible (UV–Vis) spectra were collected on a Shimadzu UV–3600 spectrometer. Neutron diffraction tests were conducted on the BT–1 neutron powder diffractometer at the National Institute of Standards and Technology (NIST) Center for Neutron Research. Field–emission transmission electron microscopy (FE–TEM) was conducted on a JEOL JEM–2100F field emission electron microscope equipped with an energy dispersive X–ray spectrometer (EDX, Oxford Instruments, 80 mm^2 detector). Field–emission scanning electron microscopy (FE–SEM) was conducted on a JEOL JSM–7610F scanning electron microscope equipped with an EDX (Oxford Instruments, Model 7426). Each sample was treated via carbon sputtering for 45 s before observation. Cryogenic transmission electron microscopy (cryo–TEM) was conducted on a Titan Krios 300 keV transmission electron microscope (FEI) with a Falcon II camera. Fluorescence laser scan confocal microscopy was conducted with a Nikon A1plus camera and Ti microscope. Confocal images were acquired with NIS-Elements C package. Ratiometric fluorescence spectra were collected at room temperature on a Photon Technology International/QuantaMaster 800 (PTI/QM, USA) spectrometer. Optical and fluorescence microscopy images were acquired using a Nikon Ti–U fluorescence microscope with Nikon DS-Ri2 camera. Stopped–flow measurements were conducted on a Chirascan SX20 stopped–flow spectrometer (Applied Photophysics). Dynamic light scattering was conducted using a NanoBrook ZetaPlus particle electrophoresis system (Brookhaven Instruments). Contact angle goniometry was conducted on a telescopic goniometer (Rame–Hart, Model 100–00–(230)). Solid atomic force microscopy (AFM) was conducted on Bruker Dimension ICON with Nanoscope V controller using tapping mode. The solid AFM data were processed with Nanoscope 9.7 and NanoScope Analysis 2.0. Liquid AFM was conducted on ParkSystems (Suwon, South Korea) using tapping mode and analysed using Park Systems XEI 1.8. The contact angle measurements were performed on a VCA optima surface analysis system (AST Products Inc., Billerica, MA, USA) using the sessile drop method with a 0.2 μL deionised water droplet.

Synthesis of CC1. Ethyl acetate (10 mL) was used to dissolve 1,3,5–triformylbenzene (40 mg, 0.25 mmol). Then, a solution of ethylene diamine (22.2 mg, 0.373 mmol) dissolved in ethyl acetate (10 mL) was added to the 1,3,5–triformylbenzene solution. The mixture was sealed in a vial and left to stand at room temperature for 1–2 days. Needle–like crystals were formed on the wall of the vial. The crystals were washed with diethyl ether (3 × 10 mL) and dried under vacuum. MS (ES+) 793.4 ($[M+H]^+$).

Synthesis of CC3. A typical procedure is as follows: dichloromethane (3 mL) was added slowly to solid 1,3,5–triformylbenzene (40 mg, 0.25 mmol) at room temperature. Finally, a solution of (R,R)–1,2–diaminocyclohexane (40 mg, 0.35 mmol) in dichloromethane (3 mL) was added. The vial of the mixture solution was capped and left to stand for one week. The CC3 crystals

gradually grew on the wall of the vial. The crystalline product was removed by centrifugation and washed once with dichloromethane, followed by three times with ethanol and diethyl ether each. The crystals were then further dried under vacuum overnight. MS (ES+) 1117.8 ($[M+H]^+$).

Synthesis of CC3 in polar solvents. Instead of using dichloromethane, CC3 crystals can be successfully grown in polar mixture solvent consisting of tetrahydrofuran/water or ethanol/methanol/water. Generally, 1,3,5–triformylbenzene (40 mg, 0.25 mmol) was dissolved in tetrahydrofuran (2 mL) or ethanol/methanol in equal parts (2 mL), and (R,R)–1,2–diaminocyclohexane (40 mg, 0.35 mmol) was dissolved in tetrahydrofuran/water or methanol/water in equal parts (2 mL). The (R,R)–1,2–diaminocyclohexane solution was then added slowly to 1,3,5–triformylbenzene solution. The mixture was sealed in a vial and let stand for 1–2 days. Crystals were found on the wall of the vial. CC3 tends to form small and uniform crystals. The usage of methanol in the solvent generally results in twinned crystals.

Synthesis of RCC3. CC3 (250 mg, 0.22 mmol) was dissolved in a dichloromethane/methanol mixture (1:1 v/v, 50 mL) by stirring. When this solution became clear, excess sodium borohydride (1.00 g, 26.5 mmol) was added and the reaction was stirred for a further 24 h at room temperature. The solvent was removed under reduced vacuum using a rotary evaporator until a gel–like product was obtained. The off–white solid was extracted with dichloromethane (20 mL) and washed with water (2 × 100 mL). The dichloromethane phase was evaporated under vacuum. RCC3 was obtained as an off–white solid. MS (ES+) 1155.9 ($[M+H]^+$).

Synthesis of FT–RCC3. Paraformaldehyde (20 mg) dissolved in methanol (10 mL) was stirred at 70 °C. RCC3 (30 mg, 0.026 mmol) dissolved in methanol (10 mL) was then added to the paraformaldehyde solution. A white precipitate appeared upon addition of RCC3. The reaction was stirred for a further 2 h at 70 °C before allowing to cool down to room temperature. The FT–RCC3 precipitate was washed with methanol (3 × 10 mL) and dried under vacuum. MS (ES+) 1214.0 ($[M+H]^+$).

Synthesis of CC5. Tris(4–formylphenyl)amine (16.5 mg, 0.05 mmol) was dissolved in dichloromethane (2 mL), and (1S, 2S)–trans–1,2–cyclopentanediamine dihydrochloride (13 mg, 0.075 mmol) was dissolved in methanol (2 mL). Triethylamine (21 μL, 0.15 mmol) was added to (1S, 2S)–trans–1,2–cyclopentanediamine dihydrochloride solution. The (1S, 2S)–trans–1,2–cyclopentanediamine dihydrochloride solution was then added to tris(4–formylphenyl)amine solution slowly. The two solutions were immiscible at this stage. The vial was sealed and let stand for 2–3 days at room temperature until light yellow crystals were found on the wall of the vial, and the two solutions became totally miscible. The crystals were washed with aliquots of diethyl ether (3 × 10 mL) and ethanol (3 × 10 mL) before being dried under vacuum. MS (ES+) 1702.9 ($[M+H]^+$).

Synthesis of CC19. Dichloromethane (3 mL) was used to dissolve 2–hydroxy–1,3,5–benzenetricarbaldehyde (40.3 mg, 0.25 mmol). A solution of (R,R)–1,2–diaminocyclohexane (40 mg, 0.35 mmol) in dichloromethane (3 mL) was then added slowly. Orange powder was obtained almost immediately. The powder was dissolved by the further addition of dichloromethane (1 mL). The vial containing the mixture was sealed and left to stand at room temperature for 1–2 days. Orange crystals were found on the wall of the vial. The crystals were

washed with aliquots of diethyl ether (3 × 10 mL) and dried under vacuum. MS (ES+) 1181.7 ($[M+H]^+$).

Synthesis of CC19 in polar solvents. Instead of using dichloromethane, CC19 crystals were grown in polar mixture solvent consisting of ethanol/methanol/water. Generally, 2–hydroxy–1,3,5–benzenetricarbaldehyde (40.3 mg, 0.25 mmol) was suspended in ethanol/methanol in equal parts (2 mL), and (R,R)–1,2–diaminocyclohexane (40 mg, 0.35 mmol) was dissolved in methanol/water in equal parts (2 mL). The (R,R)–1,2–diaminocyclohexane solution was then added slowly to 1,3,5–triformylbenzene solution. The mixture was sealed in a vial and let stand for overnight. Crystals were found on the wall of the vial. CC19 tends to form small and uniform yellow crystals.

Synthesis of Pd@RCC3. RCC3 (10 mg, 0.0087 mmol) was dispersed in deionised water (5 mL) by stirring for 2 h. Then palladium acetate (26 µL, 0.25 mmol) dissolved in dichloromethane (10 $mgmL^{-1}$) was added to RCC3 solution. The mixture was stirred for another 2 h before sodium borohydride (30 µL, 0.0079 mmol) dissolved in water (10 $mgmL^{-1}$) was added. The mixture was centrifuged and washed with water 5 times. The wet Pd@RCC3 crystals were dried under a reduced pressure at 75 °C overnight. The dried Pd@RCC3 was then re–dissolved in dichloromethane. Pd@RCC3 was recrystallized by slow evaporation under room temperature.

Synthesis of ASPOC. (R,R)–1,2–diaminocyclohexane (52.8 mg, 0.46 mmol) and ethylene diamine (27.8 mg, 0.46 mmol) were dissolved in dichloromethane (3 mL). This solution was then slowly added to 1,3,5–triformylbenzene (100 mg, 0.62 mmol) dissolved in dichloromethane (10 mL). The mixture was sealed in a vial and left to stand at room temperature for 7 days. The mixture was dried by solvent evaporation using rotary evaporator under reduced pressure and washed five times with aliquots of ethyl acetate.

Estimation of embedding efficiency of RCC3 in liposome. The embedding efficiency of RCC3 in liposome was measured using UV–Vis spectroscopy. The embedding efficiency of the channels was calculated based on the calibration curves of the UV–Vis absorbance of blank liposomes and RCC3 dissolved in methanol. Briefly, stock RCC3 (1 $mgmL^{-1}$) in methanol was added to plain liposome solution (200 mL of 1 $mgmL^{-1}$ liposome solution in HEPES buffer) at various fmCLRs (0.01 to 0.05). The calibration curve was constructed by matching the absorbance intensity with the fmCLR of the samples. A wavelength of 298.5 nm was chosen.

Sample preparation and image acquisition of Cryo–TEM. Liposomes containing Pd@RCC3 were prepared using egg–yolk phosphatidylcholine (EYPC) and 1,2–dioleoyl–sn–glycero–3–phospho–L–serine (DOPS) in a mole ratio of 4:1 using the reverse–phase method. EYPC (79.2 µL, 10 mg mL^{-1})/DOPS (20.8 µL, 10 mg mL^{-1}) in chloroform and 0.03 fmCLR Pd@RCC3 dissolved in chloroform were added to a round–bottom flask. Chloroform, diethyl ether, and HEPES buffer were then added in the same flask in a volume ratio of 2:1:1. The flask was kept degassed under dry argon. Subsequently, the mixture was sonicated at 0–4 °C until a homogeneous water–in–oil mixture was obtained. The organic solvents were then removed under a reduced pressure using a rotary evaporator (178 rpm, 45 °C, in air). The liposomes obtained were extruded through a hand–held extruder with 0.2 µm track–etched membrane for 21 times to obtain monodisperse, unilamellar vesicles. The cryo–TEM images were recorded at a nominal

magnification of 59,000× giving a pixel size of 1.43 Å at the specimen. The images were acquired under high dosage of 50 e^{-} $Å^{-2}$.

Sample preparation of POC–coated porous anodic aluminum oxide (AAO) substrate. POCs were coated on AAO substrates by spin–coating. POC solutions (100 μL, 1 wt% POC in dichloromethane (< 2 wt% methanol was added to increase solubility of CC3, CC19, and FT–RCC3)) were added to fully cover the AAO substrate before coating (1000 rpm, 1min). CC5 has very limited solubility (ca. 0.0002 wt% was used), hence multiple coating was conducted. The POC–coated substrates were kept under vacuum overnight to remove residual organic solvents before testing.

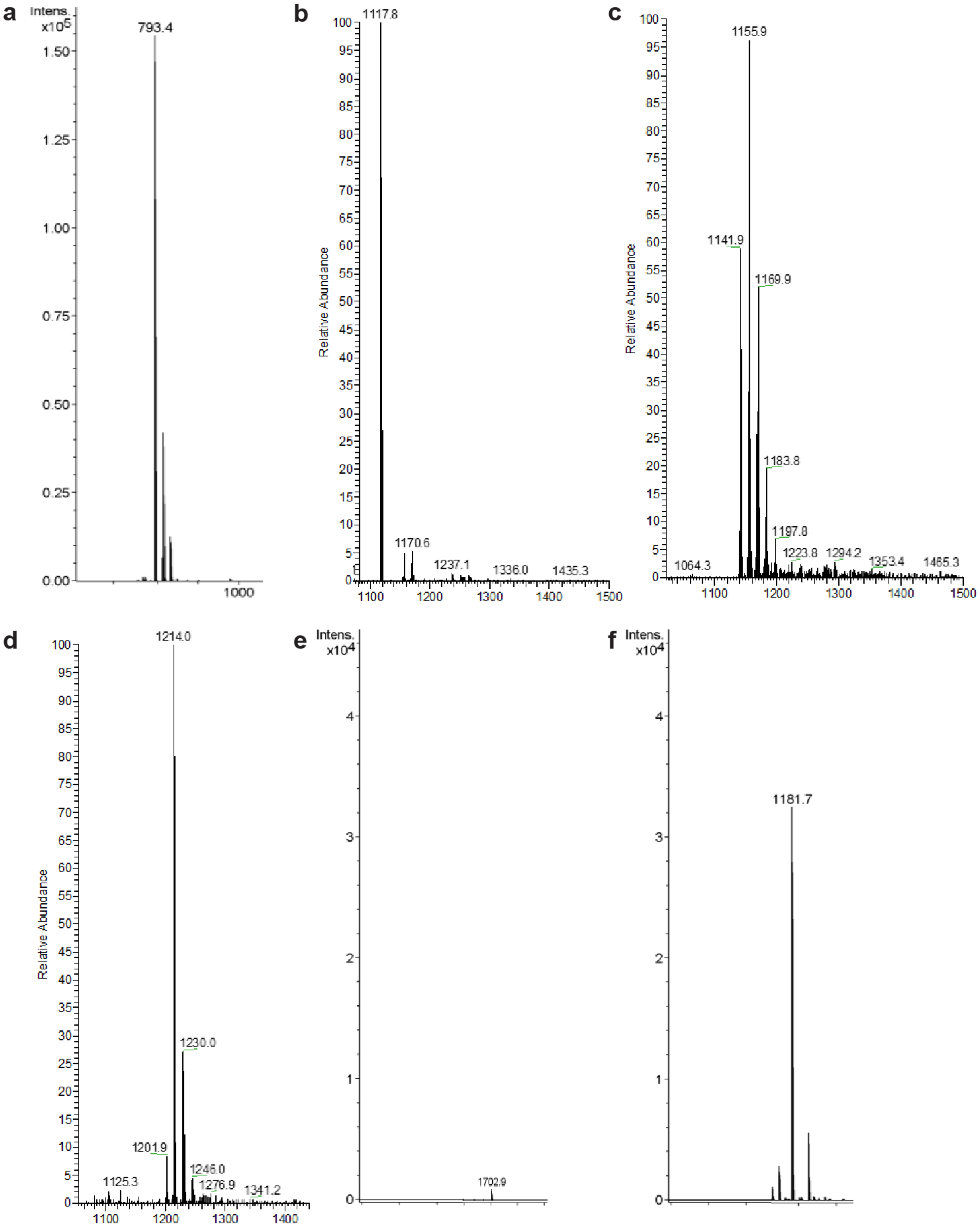

Supplementary Figure 1 | Mass spectrometry data of POCs. (**a**) CC1. (**b**) CC3. (**c**) RCC3. (**d**) FT–RCC3. (**e**) CC5. (**f**) CC19.

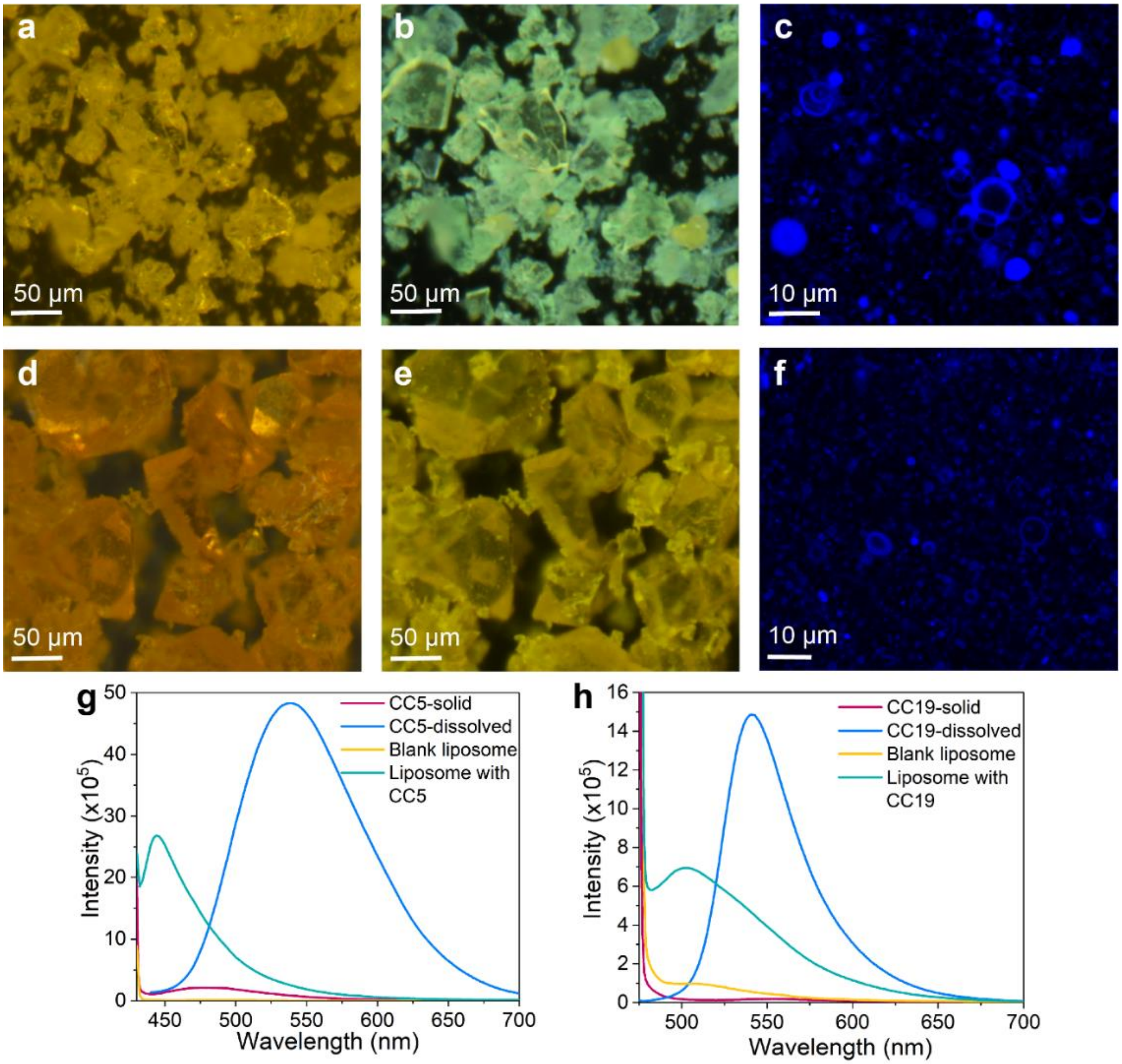

Supplementary Figure 2 │ Fluorescence microscopy and spectroscopy of CC5 and CC19. CC5 crystals under white light (**a**) or at an excitation of 375 nm (**b**). (**c**) Confocal microscopy of liposomes containing CC5 at an excitation of 402 nm. CC19 crystals under white light (**d**) or at an excitation of 375 nm (**e**). (**f**) Confocal microscopy of liposomes containing CC19 at an excitation of 402 nm. (**g**) Spectra of CC5 (solid or dissolved in chloroform) and liposome containing CC5 (fmCLR of 0.035) at an excitation of 421 nm. (**h**) Spectra of CC19 (solid or dissolved in chloroform) and liposome containing CC19 (fmCLR of 0.07) at an excitation of 466 nm.

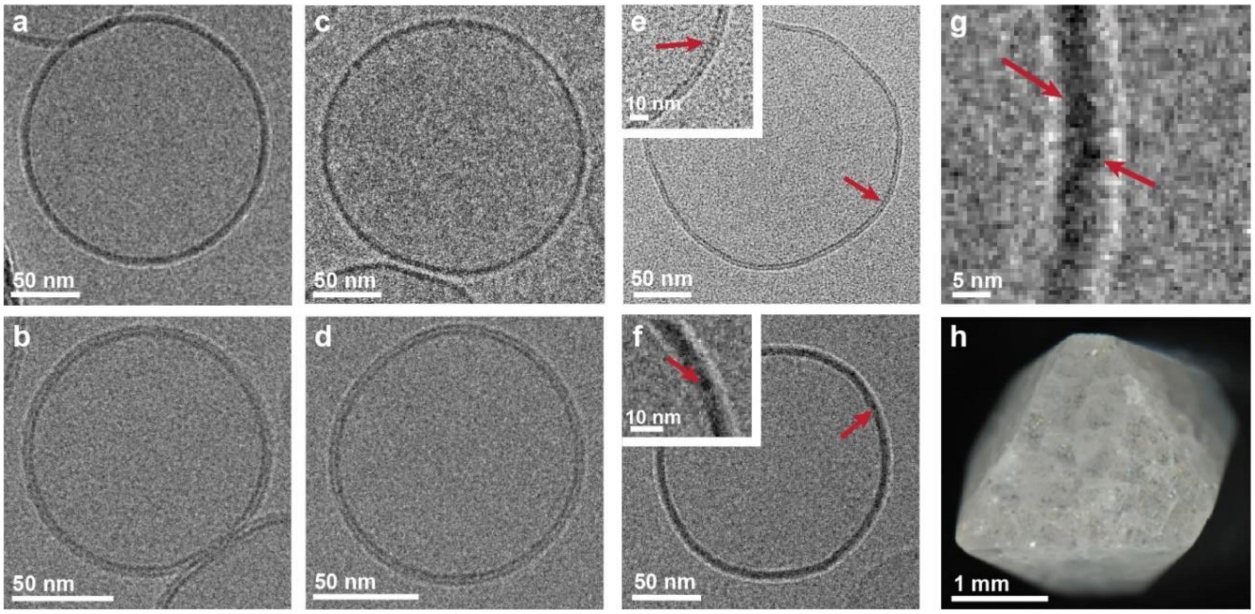

Supplementary Figure 3 | Cryo–TEM images and optical images of liposome vesicles and CC3 single crystal. (**a**) Cryo–TEM image of blank liposome under low resolution and high contrast. (**b**) Cryo–TEM image of blank liposome under high resolution and low contrast. (**c**) Cryo–TEM image of liposome with CC3 under low resolution and high contrast. (**d**) Cryo–TEM image of liposome with CC3 under high resolution and low contrast. (**e**) Cryo–TEM image of liposome embedded with Pd@RCC3 obtained at high resolution. (**f**) Cryo–TEM image of liposome embedded with Pd@RCC3 obtained at high contrast showing dark tetrahedral–shaped object in the lipid bilayer. Insert shows the enlarged part of Pd@RCC3 inside lipid bilayer. Red arrows indicate the positions of Pd@RCC3. (**g**) Enlarged image (under low resolution and high contrast) of cryo–TEM of liposome with Pd@RCC3 showing dark diamond–shaped objects (positions shown by red arrows). (**h**) Optical image of one CC3 single crystal under white light.

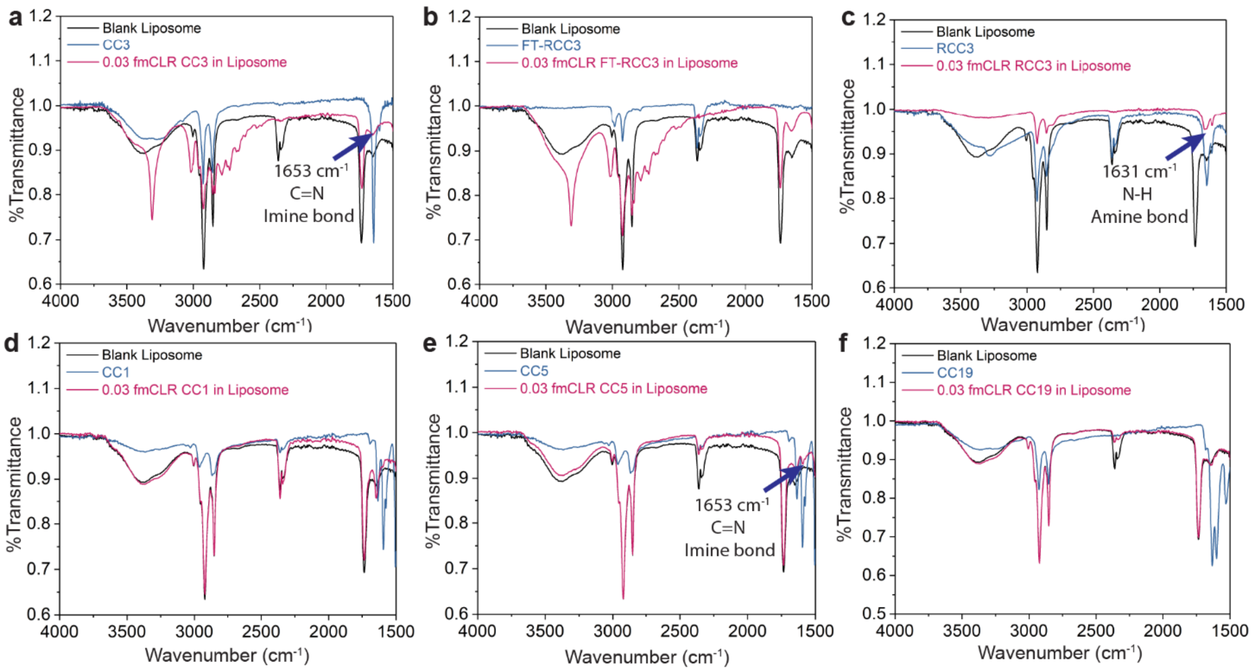

Supplementary Figure 4 | FTIR spectra of POCs in liposomes. (**a**) CC3 in liposome, (**b**) FT–RCC3 in liposome, (**c**) RCC3 in liposome, (**d**) CC1 in liposome, (**e**) CC5 in liposome, and (**f**) CC19 in liposome, in comparison to the blank liposome and pure POCs. Imine bond (C=N) peaks at 1653 cm^{-1} were observed for liposome samples incorporated with imine cages, i.e., CC3 and CC5. Imine bond in RCC3 was reduced to amide bond (C–N) and the corresponding peak at 1653 cm^{-1} was observed for liposome incorporated with RCC3.

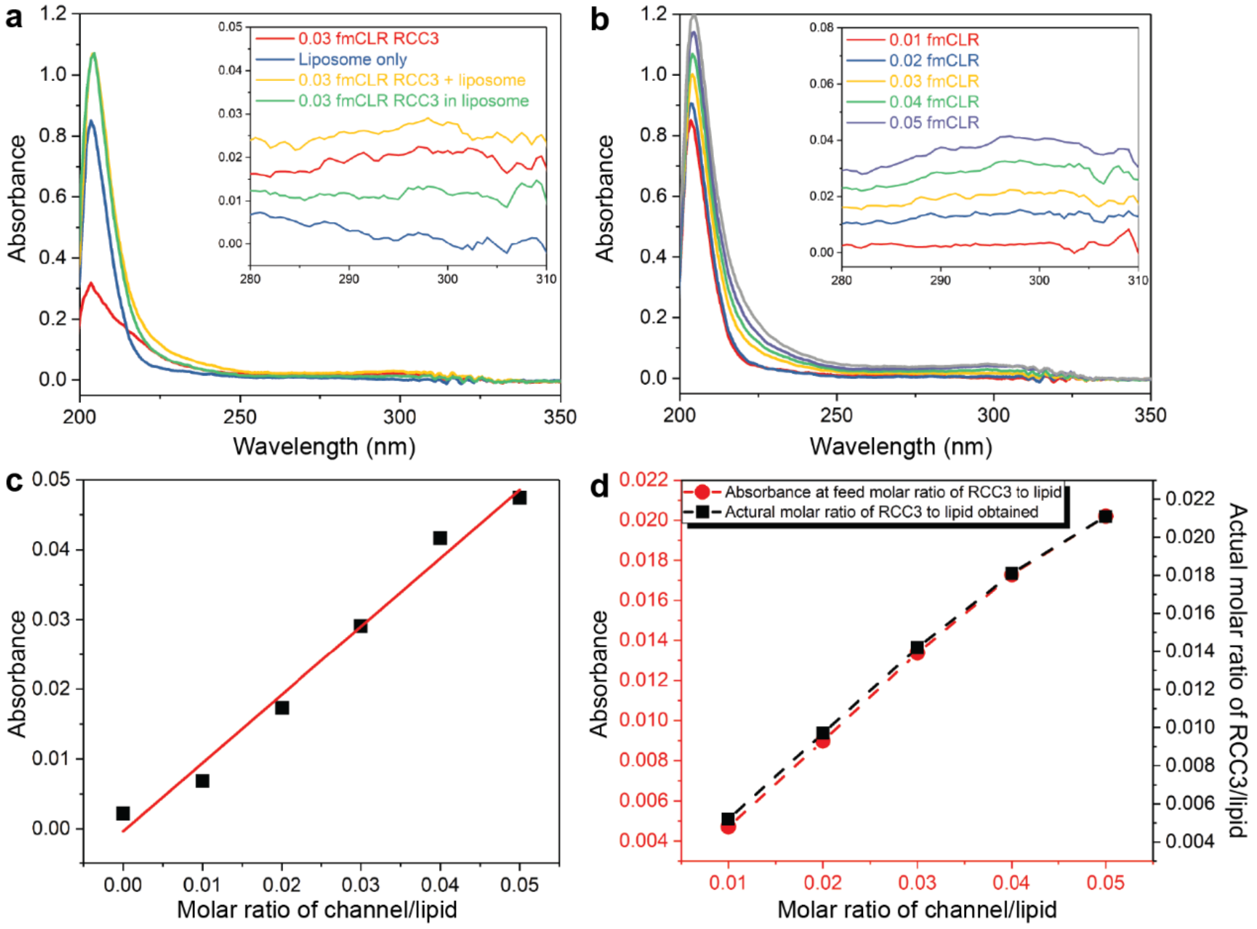

Supplementary Figure 5 | Determination of actual loading of RCC3 in liposome using UV–Vis spectroscopy. (**a**) UV–Vis spectra of RCC3 and liposome embedded with RCC3 for the determination of the absorption wavelength. The characteristic hump was observed for RCC3 at 298 nm (insert, red curve). (**b**) Raw data for the construction of the standard curve. The sample solution was topped up with methanol to 2 mL. Characteristic peaks at 298 nm were observed to increase in absorbance intensity with increasing fmCLR. (**c**) The standard curve for RCC3 in liposome. (**d**) Actual embedding of samples of RCC3 in liposome for fresh samples of liposomes embedded with RCC3 of various fmCLRs.

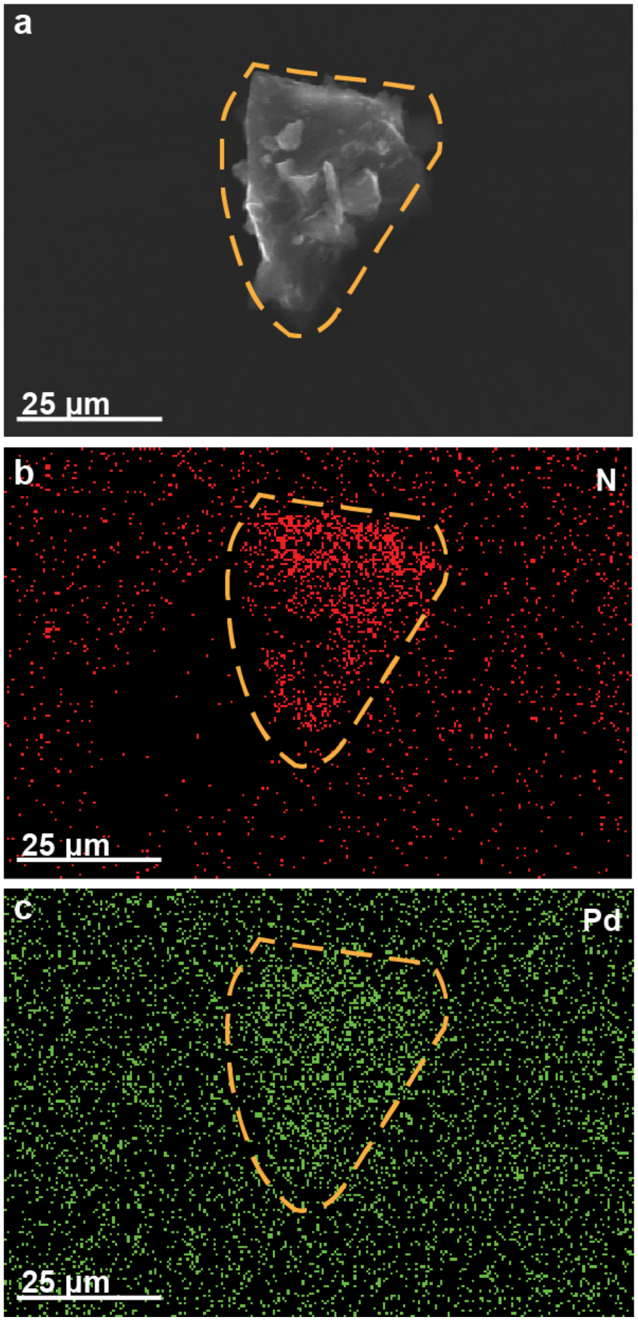

Supplementary Figure 6 │ SEM image and EDX spectroscopy of Pd@RCC3. (**a**) SEM image of Pd@RCC3. EDX mapping of nitrogen (**b**) and palladium (**c**) elements. Note: despite the large background noise, a clear outline of the Pd@RCC3 can still be seen in the EDX mapping of palladium.

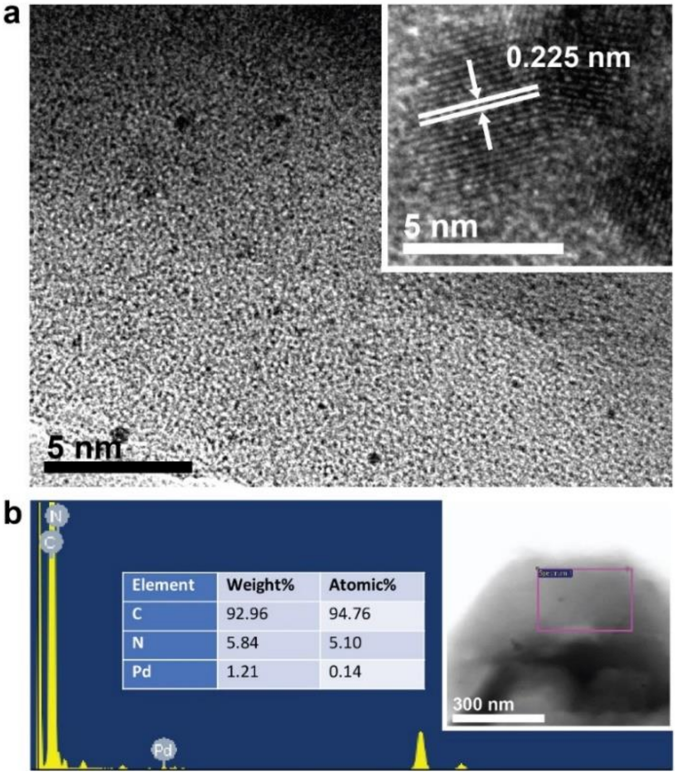

Supplementary Figure 7 │ TEM image and EDX spectroscopy of Pd@RCC3. (**a**) TEM image of Pd@RCC3. Insert: lattice of Pd nanoparticles. (**b**) TEM–EDX spectroscopy of Pd@RCC3 identifying the percentage composition of elements at the specific boxed area in the insert TEM image.

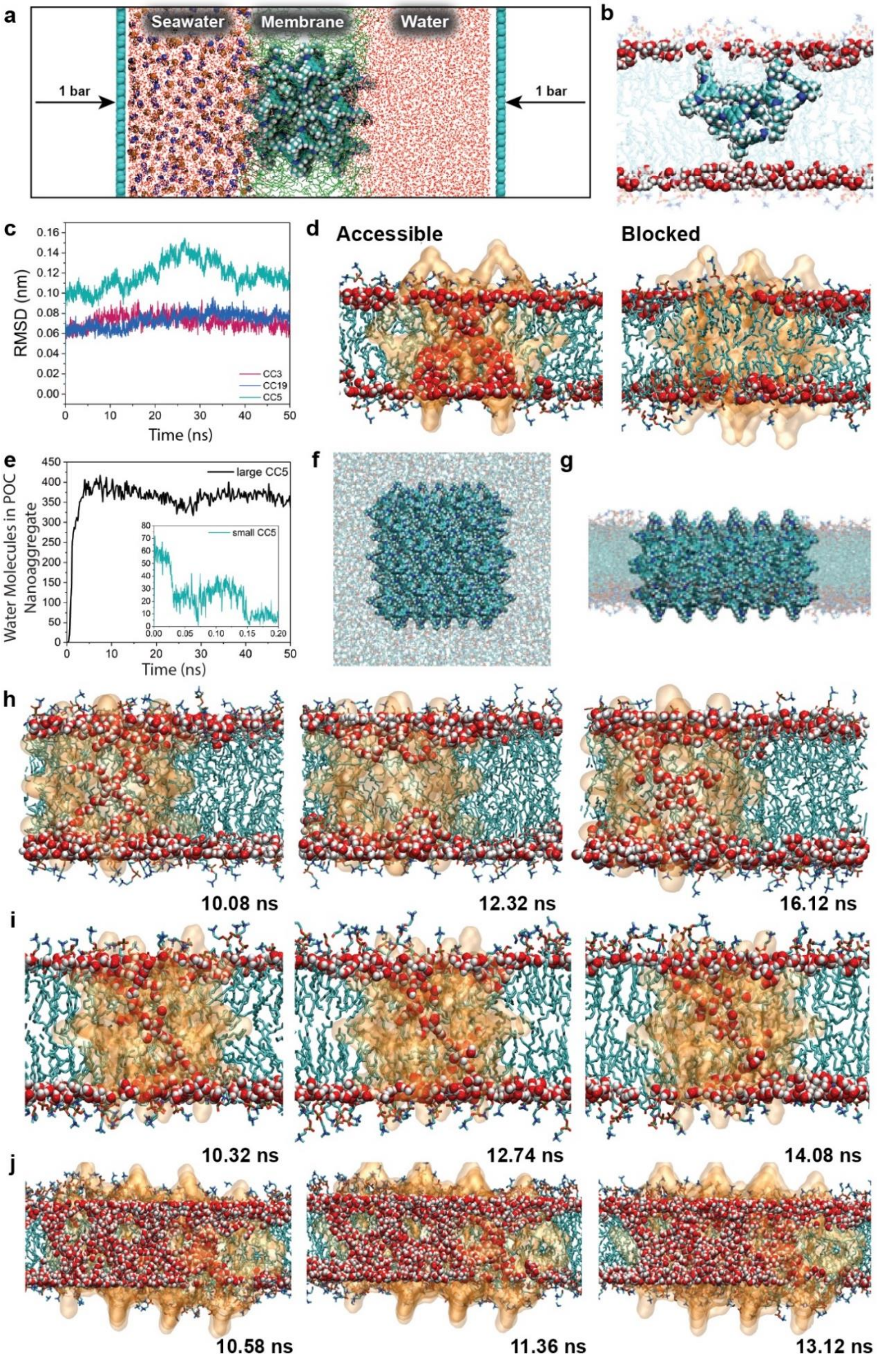
a
Seawater
Membrane
Water
1 bar
1 bar
b
c
RMSD (nm)
Time (ns)
CC3
CC19
CC5
d
Accessible
Blocked
e
Water Molecules in POC Nanoaggregate
Time (ns)
large CC5
small CC5
f
g
h
10.08 ns
12.32 ns
16.12 ns
i
10.32 ns
12.74 ns
14.08 ns
j
10.58 ns
11.36 ns
13.12 ns

Supplementary Figure 8 | Molecular dynamics (MD) simulation of water permeation. (**a**) A representative simulation system, in which a CC3 nanocrystal with a dimension of 4.73 × 4.73 × 4.73 nm^3 was embedded in the 1–palmitoyl–2–oleoyl phosphatidylcholine (POPC) lipid membrane. Two chambers (2 M NaCl aqueous solution on the left and pure water on the right) were separated by the membrane. Two graphene pistons were placed outside the chambers and exerted by atmospheric pressure (1 bar). Colour codes: CC3 crystal, white–cyan spheres; POPC, green; Na^+, orange; Cl^-, blue; H_2O, red–white spheres; graphene layers, cyan. (**b**) A CC3 nanoaggregate (3 CC3 molecules) in the lipid membrane. Note that no water permeation was observed in this case. (**c**) Root–mean–square deviation (RMSD) evolution of POCs versus time. Note that CC3, CC19 and CC5 nanoaggregates remain stable inside the lipid bilayer. (**d**) Illustration of the small CC5 nanoaggregate (17 cages) quickly blocked by lipid tails during simulation. (**e**) Wetting–dewetting profile in large CC5 nanoaggregate (75 cages). Insert: wetting–dewetting profile in small CC5 nanoaggregate (17 cages). (**f**) Top view of a large CC5 nanoaggregate. (**g**) Side view of a large CC5 nanoaggregate. (**h**–**j**) Simulation snapshots at various time intervals for CC3 nanoaggregate, CC19 nanoaggregate, and large CC5 nanoaggregate, respectively. Note that CC3 displays a wetting–dewetting transition while CC5 and CC19 do not.

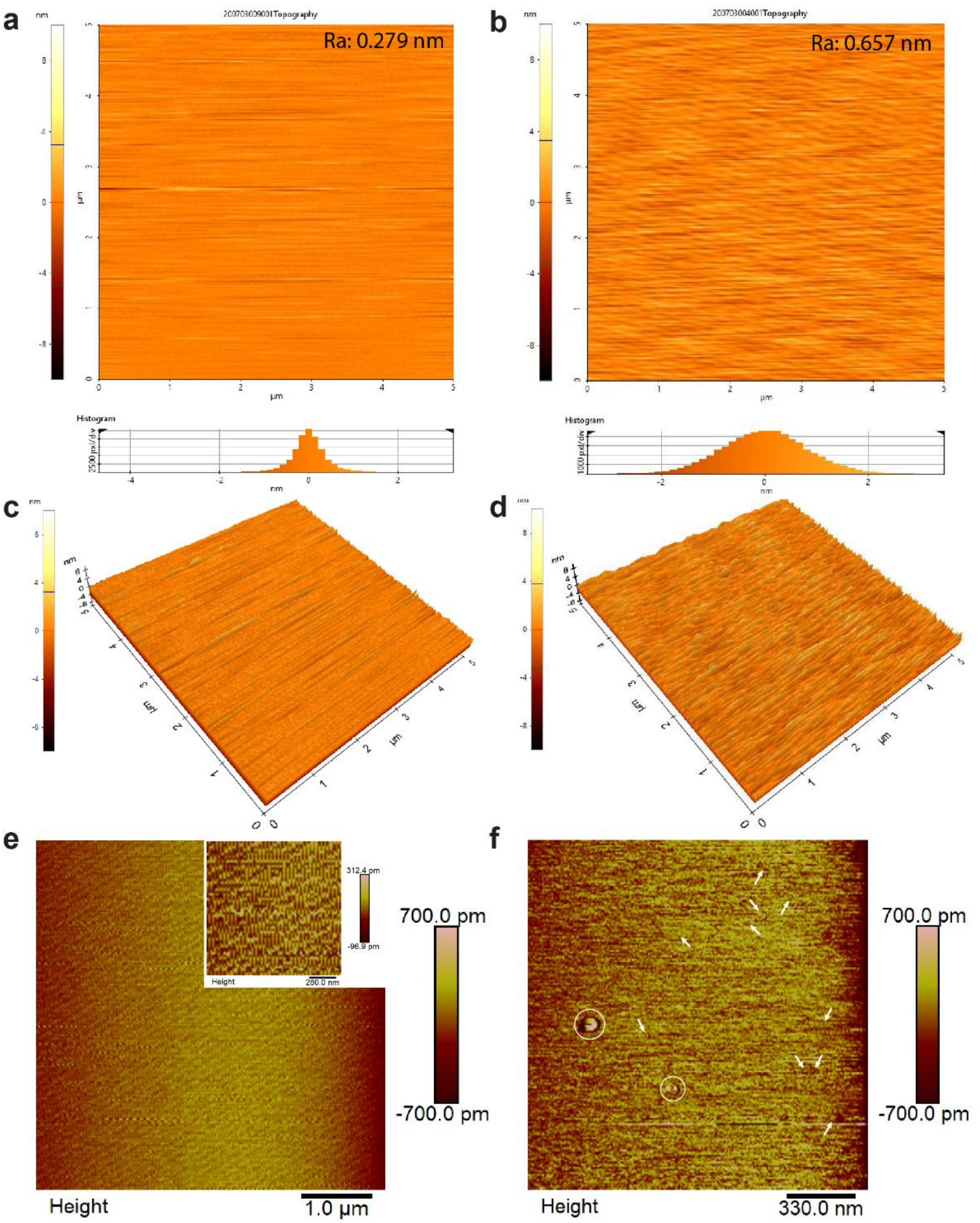

Supplementary Figure 9 | AFM images of blank liposome and liposome with CC3. 2D AFM images of blank supported lipid bilayer (SLB) (**a**) and CC3–incorporated (fmCLR 0.03) SLB (**b**) on mica sheet observed using tapping mode in buffer (10 mM HEPES). The same images were observed in 3D showing blank SLB (**c**) being smoother compared to CC3–incorporated (fmCLR 0.03) SLB (**d**) on mica sheet. Solid AFM was performed in tapping mode for blank SLB (**e**) and CC3–incorporated (fmCLR 0.03) SLB (**f**) on mica sheet. Some weak protrusions were observed in CC3–incorporated SLB and they are marked with white arrows. Larger CC3 nanoaggregates deposited on the SLB (circled in white) were also observed. **Note:** Solid SLB appears regularly patterned due to the smoothness of blank lipid bilayer and system noise.

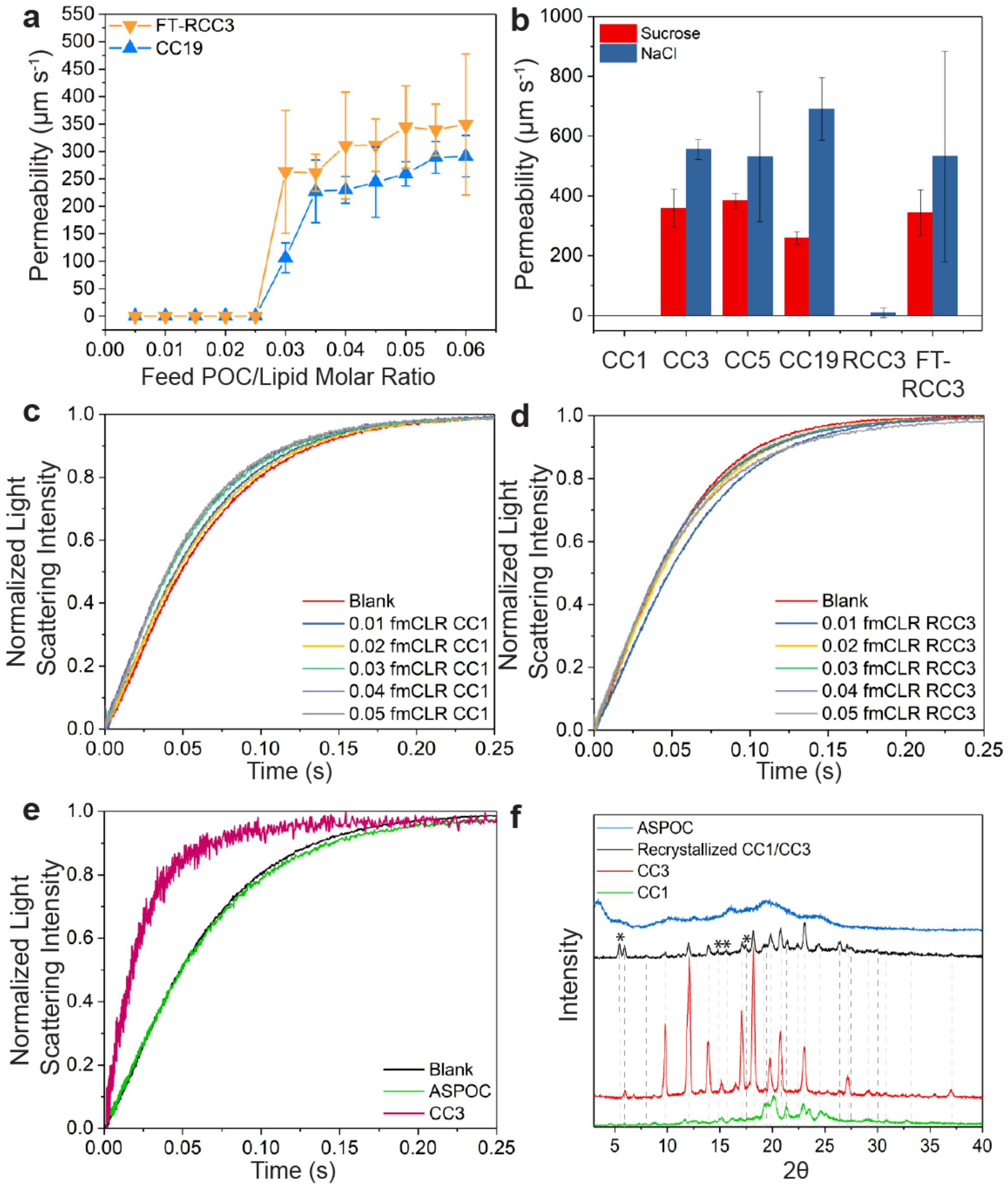

Supplementary Figure 10 | Water permeability of POCs. (**a**) Water permeability of liposomes with increasing feed loading of CC19 and FT–RCC3. (**b**) Water permeabilities of liposomes with various POCs (CC1 (fmCLR 0.03), CC3 (fmCLR0.03), RCC3 (fmCLR 0.03), FT–RCC3 (fmCLR 0.06), and CC19 (fmCLR 0.06)) when exposed to sucrose and sodium chloride environment. Stopped–flow light–scattering raw data for CC1 (**c**) and RCC3 (**d**) showing negligible water permeation improvement with increasing loading compared to blank liposome. (**e**) Stopped–flow light–scattering raw data of equal mass loading of CC3 (fmCLR 0.03) and ASPOC. (**f**) Powder X–ray diffraction of crystalline CC1 and CC3, ASPOC, as well as recrystallized equal molar CC1 and CC3. New peaks of recrystallized CC1/CC3 are marked with asterisk (*). Error bars represent standard deviation of three independent replicates.

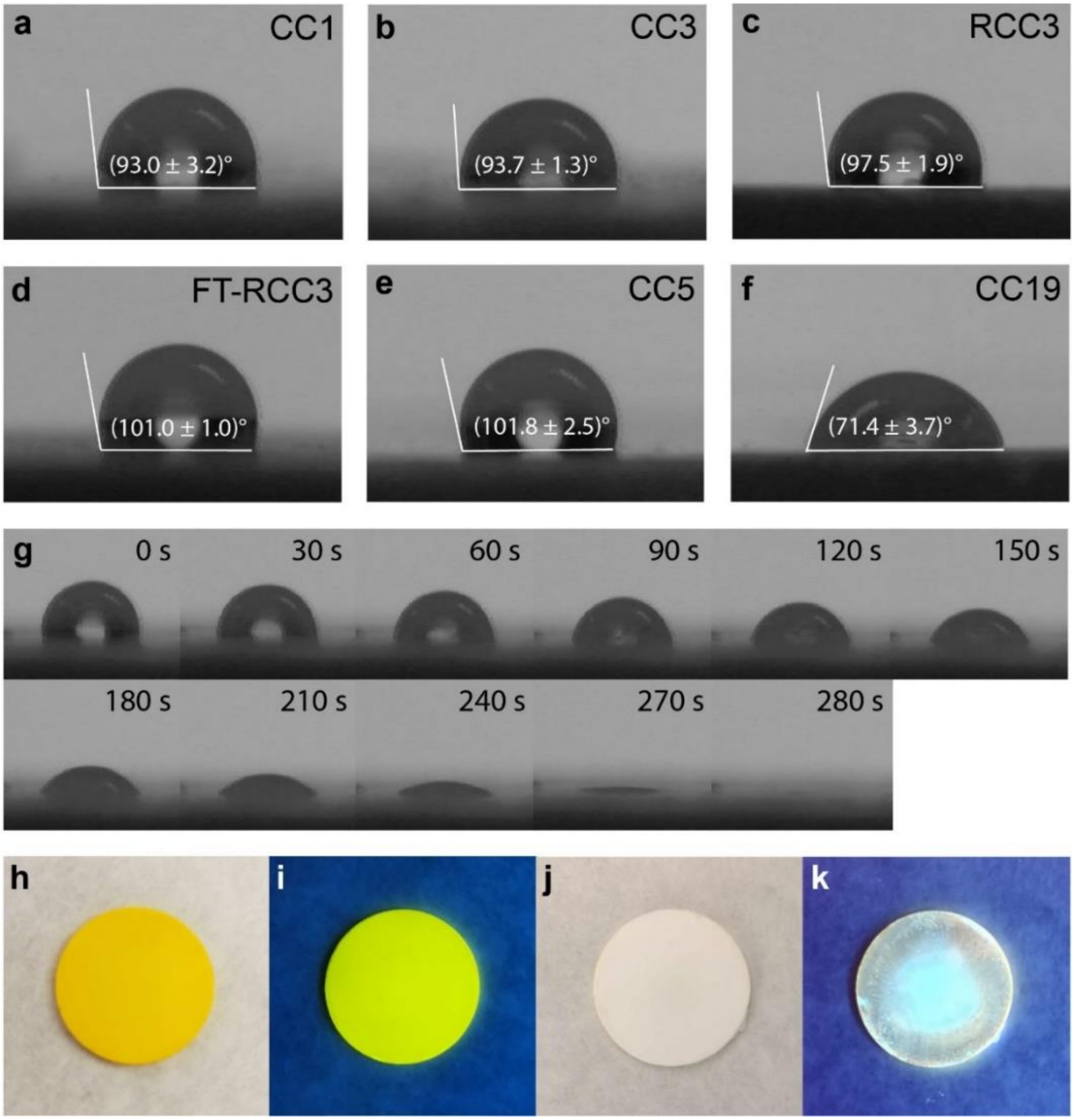

Supplementary Figure 11│Contact angles of POCs. (**a**) CC1, (**b**) CC3, (**c**) RCC3, (**d**) FT–RCC3, (**e**) CC5, and (**f**) CC19 coated on anodized aluminum oxide (AAO) substrate. (**g**) Time trace of a water drop (0.2 μL) on AAO substrate spin–coated with CC3 under enclosed environment. (**h**–**i**) AAO substrate coated with CC19 observed under white light (**h**) and UV light (365 nm, **i**) respectively. (**j**–**k**) AAO substrate coated with CC5 observed under white light (**j**) and UV light (365 nm, **k**) respectively. Note that CC5 was difficult to be coated evenly due to its limited solubility in solvents that resulted in a very diluted doping solution. Therefore, the sample shown in (**j**–**k**) was obtained after 5 times coating. Error bars present standard deviation of three readings.

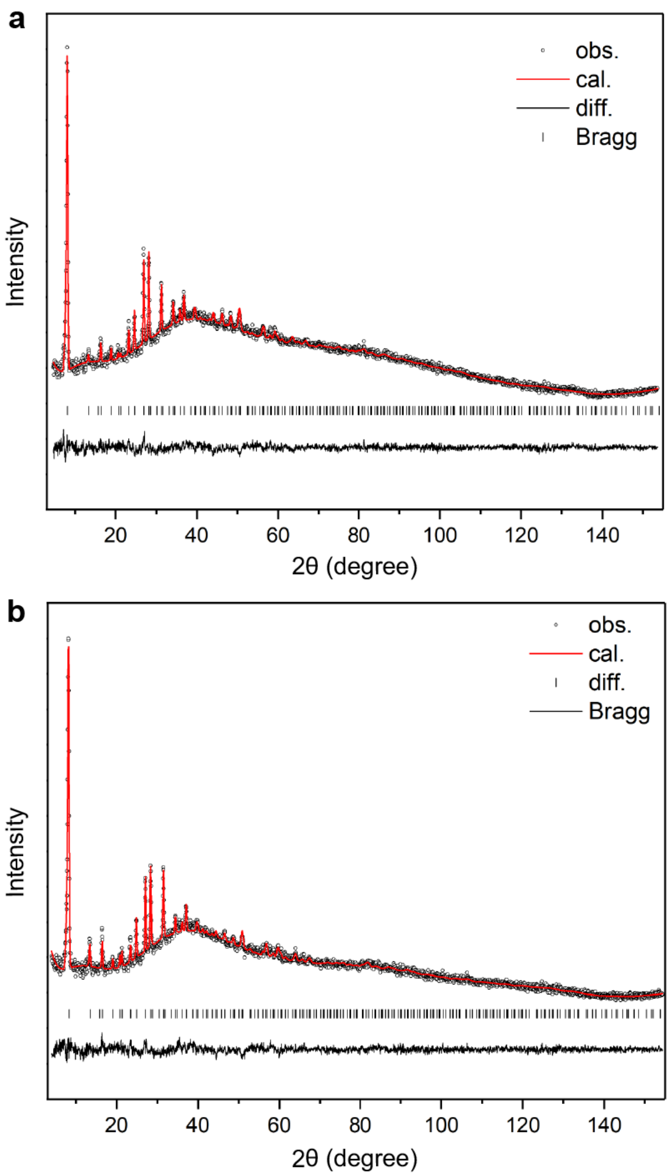

Supplementary Figure 12 | Rietveld refinements of the neutron powder diffraction data. (**a**) CC3 with D_2O and (**b**) CC19 with D_2O, measured at 296 K. The broad background in the data is due to the diffuse scattering from the excess liquid–phase D_2O presented along with the cage crystals in the samples.

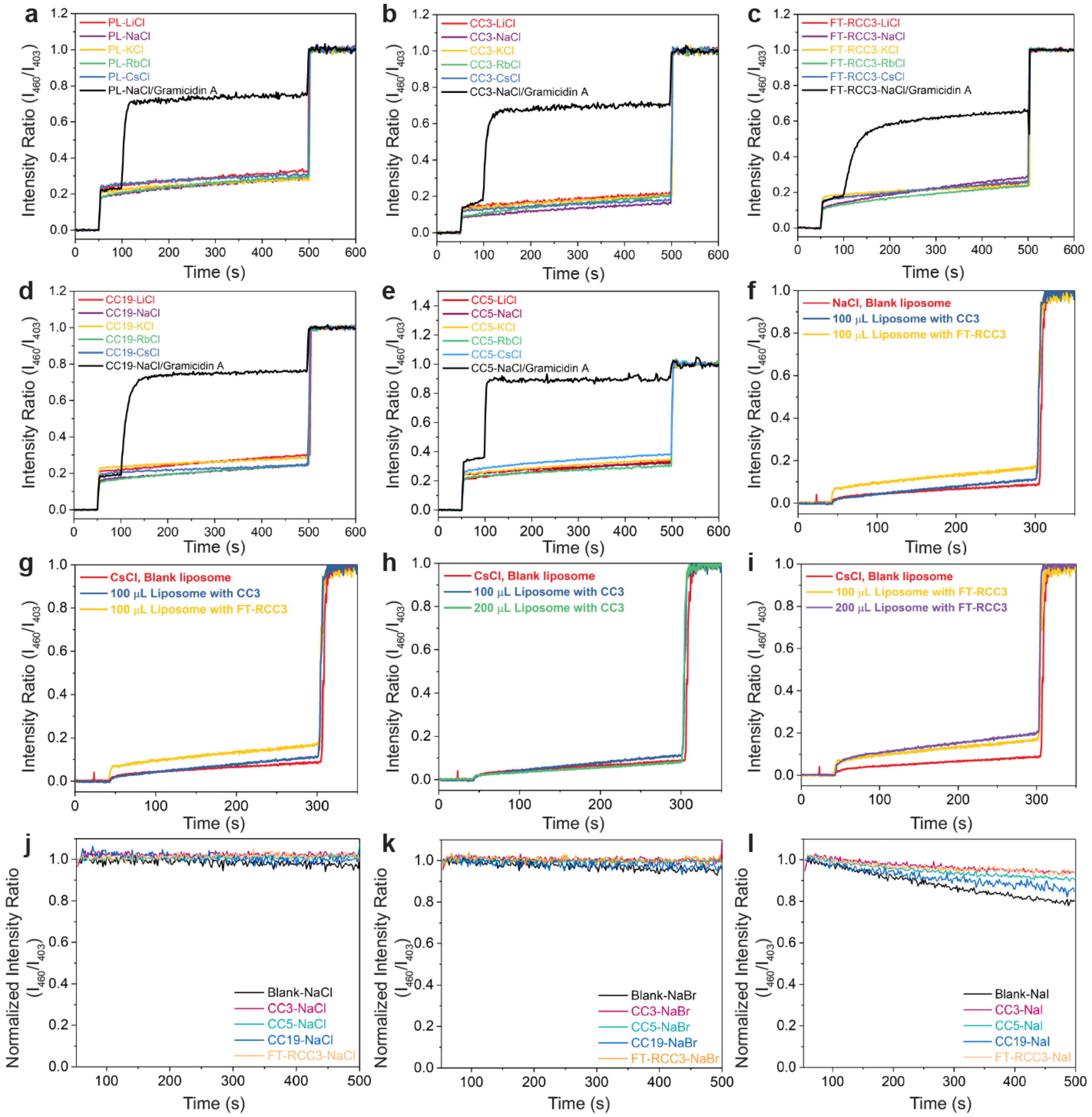

Supplementary Figure 13 │ Ratiometric measurement data for the determination of cation transport through the studied POCs. Normalized fluorescence intensity ratio (I_{460}/I_{403}) of pyranine emission at 510 nm for cation (Li^+, Na^+, K^+, Rb^+, Cs^+) transport in POC–embedded large unilamellar vesicles (LUVs, fmCLR = 0.03): (**a**) Blank liposome, (**b**) CC3–embedded LUV, (**c**) FT–RCC3–embedded LUV, (**d**) CC19–embedded LUV, (**e**) CC5–embedded LUV, (**f**) Na^+ transport through CC3 and FT–RCC3, (**g**) Cs^+ transport through CC3 and FT–RCC3, (**h**) Cs^+ transport in various volumes of liposome with CC3, (**i**) Cs^+ transport in various volumes of liposome with FT–RCC3. Normalized fluorescence intensity ratio (I_{460}/I_{403}) of pyranine emission at 510 nm for anion (Cl^-, Br^-, I^-) transport in POC–embedded LUVs (fmCLR = 0.03): (**j**) NaCl, (**k**) NaBr, (**l**) NaI.

Note: For the cation transport tests, liposomes were suspended in buffer solution at pH 7. At 50 s, a base pulse (additional of NaOH) induced a change in extravesicular environment. This induces the efflux of protons from the liposome, deprotonating the pyranine and results in an increase in the fluorescence intensity ratio. We did not observe significant increase in fluorescence intensity ratio in both blank liposome and liposome containing POCs. Gramicidin A dissolved in dimethyl sulfoxide (265 nM) was added to each sample at 100 s inducing large increase in fluorescence intensity ratio, which indicates significant cation transport. At the end of the experiment, detergent was added to completely destroy the liposomes and release all pyranine, which is shown as a sudden spike in fluorescence intensity ratio. Ratiometric results have been confirmed by Dr. Mihail Barboiu's group (figures **f–i**). Samples of liposomes with CC3 and FT–RCC3 were prepared accordingly: FT–RCC3 (0.5 mg, 0.00041 mmol) was evenly premixed with EYPC (10 mg, 0.013 mmol) in chloroform before evaporation; CC3 (0.26 mg, 0.00023 mmol) was evenly premixed with EYPC (10 mg, 0.013 mmol) in chloroform before evaporation. Liposomes were prepared in buffer A (100 mM NaCl, 10 μM pyranine, 10 mM phosphate buffer at pH 6.4) and then exposed to buffer B (either 100 mM NaCl or CsCl, 10 mM phosphate buffer at pH 6.4) during ratiometric measurements.

Supplementary Table 1. Calculation of POC channel permeability

POC	Critical Loading (fmCLR)	Peak Loading (fmCLR)	Highest Overall Permeability (μm s^{-1})	Assumed number of POCs per single nanoaggregate	Single channel permeability (× 10^{-14} cm^3 s^{-1})	Single channel permeability (× 10^9 water molecules s^{-1})
CC3	0.015	0.03	359 (± 63.2)	17	8.54 (± 1.50)	2.85 (± 0.50)
CC5	0.015	0.025	389 (± 49.9)	75	48.0 (± 6.16)	16.0 (± 2.06)
				17	11.1 (± 1.43)	3.71 (± 0.48)
CC19	0.03	0.06	291 (± 37.7)	17	3.79 (± 0.49)	1.27 (± 0.16)
FT–RCC3	0.03	0.055	340 (± 46.8)	17	4.74 (± 0.65)	1.58 (± 0.22)

The calculation of single channel permeability is based on equations [1], [3], and [4] in the main text assuming 17 or 75 POC molecules per nanoaggregate. Error bars represent standard deviation of three independent replicates.

Supplementary Table 2. Corrected calculation of POC channel permeability

POC	Peak Loading (fmCLR)	Highest Overall Permeability (μm s^{-1})	Assumed number of POCs per single nanoaggregate	Single channel permeability (× 10^{-14} cm^3 s^{-1})	Single channel permeability (× 10^9 water molecules s^{-1})
CC3	0.03	135 (± 23.7)	17	3.20 (± 0.56)	1.07 (± 0.19)
CC5	0.025	146 (± 18.7)	75	18.0 (± 2.31)	6.02 (± 0.77)
			17	4.17 (± 0.54)	1.39 (± 0.18)
CC19	0.06	109 (± 14.1)	17	1.42 (± 0.18)	0.48 (± 0.06)
FT–RCC3	0.055	127 (± 17.6)	17	1.78 (± 0.25)	0.59 (± 0.08)

The calculation of single channel permeability is based on equations [2], [3], and [4] in the main text assuming 17 or 75 POC molecules per nanoaggregate. Error bars represent standard deviation of three independent replicates.

Supplementary Table 3. Simulated salt rejection

Membrane configuration	Ion Rejection		
	CC3	CC5	CC19
Pure POC crystalline membrane	Yes	2 Na^+ and 2 Cl^- (after 200 ns)	Yes
Small POC nanoaggregate (17 POC molecules) in POPC bilayer	Yes	Yes	Yes
Large POC nanoaggregate (75 POC molecules) in POPC bilayer	–	1 Na^+ (after 100 ns)	–

Only CC5 showed some ion permeability, which can be attributed to its large pore size. No ion rejection was observed in small CC5 nanoaggregate before the channels were totally blocked by lipid tails.

Supplementary Table 4. Blank liposome permeability

Osmolyte	Filter size (nm)	Liposome size (nm)	k (s^{-1})	P_f (μm s^{-1})	Corrected P_f (μm s^{-1})
Sucrose	200	199.2 ± 5.0	13.4 ± 0.1	123.5 ± 2.5	46.3 ± 0.9
Sucrose	100	150.5 ± 1.1	25.5 ± 3.8	174.8 ± 8.5	65.6 ± 3.2
NaCl	200	198.6 ± 4.9	22.7 ± 0.6	205.0 ± 11.3	76.9 ± 4.2
NaCl	100	145.8 ± 2.3	39.0 ± 6.2	290.4 ± 80.6	108.9 ± 30.2

Note: Blank liposome permeabilities were tested with different liposome sizes as well as osmolytes. NaCl gave markedly higher permeabilities compared to sucrose. The shrinkage rates were obtained from single–exponential fitting of the stopped–flow data instead of double–exponential model. When fitting blank liposome data with double–exponential model, the two rates (k_1, k_2) are either similar or only one gives meaningful data. Error bars represent standard deviation of three independent replicates.

Supplementary Table 5. Unit area permeability of aquaporin, carbon nanotube and CC3

**Channels**	**Aquaporin (Aqp1)**	**Carbon nanotube**	**CC3**
Pore diameter (Å)	2.8	15	5.8
Single–channel permeability (number of water molecules per second)	4.9×10^{9}	1.9×10^{9}	2.9×10^{9}
Packing density (number of channels per μm^{2})	8.9×10^{4}	2.5×10^{3}	4.5×10^{4}
Unit area permeability (number of water molecules per second per μm^{2})	4.4×10^{14}	4.8×10^{12}	1.3×10^{14}

The permeability and packing density of Aqp1 are obtained from Murata *et al.*[1] and Walz *et al*[2]. The permeability and packing density of CNT are obtained from Tunuguntla *et al.*[3] and Holt *et al.*[4]. The unit area permeability is the water permeability per micrometre–squared area, obtained from the product of single–channel permeability and packing density. The CC3 data were calculated based on a cross–sectional area of 22.4 nm^{2} of the nanoaggregate.

Supplementary References

1 Murata, K. et al. Structural determinants of water permeation through aquaporin–1. *Nature* **407**, 599–605 (2000).
2 Walz, T. et al. The three–dimensional structure of aquaporin–1. *Nature* **387**, 624–627(1997).
3 Tunuguntla, R. H. et al. Enhanced water permeability and tunable ion selectivity in subnanometer carbon nanotube porins. *Science* **357**, 792–796 (2017).
4 Holt, J. K. et al. Fast mass transport through sub–2–nanometer carbon nanotubes. *Science* **312**, 1034–1037 (2006).
