# Supplementary material for: Porous organic cages as synthetic water channels
Source: Nat Commun. 2020 Oct 1;11:4927. doi: 10.1038/s41467-020-18639-7 (PMC7530991; doi:10.1038/s41467-020-18639-7)
Supplement: Supplementary file 3 — Description of Additional Supplementary Files [file 41467_2020_18639_MOESM3_ESM.pdf]

**Description of Additional Supplementary Files**

File Name: Supplementary Movie 1
Description: Movement of water molecules through an irregular POC nanoaggregate containing 3 CC3 molecules in the POPC bilayer under an osmotic pressure of 2 M NaCl solution.

File Name: Supplementary Movie 2
Description: Movement of water molecules through a 5.70 nm × 5.70 nm × 5.70 nm POC nanoaggregate containing 17 CC5 molecules in the POPC bilayer under an osmotic pressure of 2 M NaCl solution.

File Name: Supplementary Movie 3
Description: Movement of water molecules through a 4.73 nm × 4.73 nm × 4.73 nm POC nanoaggregate containing 17 CC3 molecules in the POPC bilayer under an osmotic pressure of 2 M NaCl solution.

File Name: Supplementary Movie 4
Description: Movement of water molecules through a 4.73 nm × 4.73 nm × 4.73 nm POC nanoaggregate containing 17 CC19 molecules in the POPC bilayer under an osmotic pressure of 2 M NaCl solution.

File Name: Supplementary Movie 5
Description: Movement of water molecules through a 10.2 nm × 10.2 nm × 5.70 nm POC nanoaggregate containing 75 CC5 molecules in the POPC bilayer under an osmotic pressure of 2 M NaCl solution.

File Name: Supplementary Data 1
Description: Sample calculations for water permeability from the data of stopped–flow experiment.
